# Causal effects of dietary composition on multiple sclerosis risk and severity: a Mendelian randomization study

**DOI:** 10.3389/fnut.2024.1410745

**Published:** 2024-05-30

**Authors:** Haitian Nan

**Affiliations:** Department of Neurology, Xuanwu Hospital, Capital Medical University, Beijing, China

**Keywords:** dietary, multiple sclerosis, risk, severity, Mendelian randomization study

## Abstract

**Objectives:**

Observational studies have found potential associations between dietary intake and multiple sclerosis (MS). However, these associations are inconsistent, and the causal relationship remains unclear. In this study, we aim to examine the causal relationship between genetically predicted dietary composition and the risk and severity of MS using two-sample Mendelian randomization.

**Method:**

Genetic instruments for 30 different dietary compositions were extracted from large-scale genome-wide association studies (GWAS), mainly from the UK Biobank dataset. The GWAS data for MS risk and severity were obtained from the International Multiple Sclerosis Genetics Consortium. The primary analysis employed either the inverse variance weighted method or the Wald ratio method to evaluate the causal association. Several sensitivity analyses were also performed.

**Results:**

Genetically predicted higher pork intake was causally associated with an increased risk of MS (odds ratio [OR] = 6.76; *p* = 0.005), while genetically driven higher cereal intake (OR = 0.43, *p* = 0.016), vitamin C supplement (OR < 0.01; *p* = 4.34 × 10^−5^), folic acid supplement (OR < 0.01; *p* = 4.91 × 10^−71^), and fish oil supplement (OR = 0.04; *p* = 0.017) were causally linked to a decreased risk of MS. In addition, genetically predicted higher alcoholic intake (OR = 1.17; *p* = 0.041) was causally associated with an increase in MS severity, while folic acid supplement (OR < 0.01; *p* = 0.015) was causally linked to a decrease in MS severity.

**Interpretation:**

This study found that increased consumption of cereal, vitamin C, folic acid, and fish oil, coupled with reduced pork and alcohol intake, may potentially decrease the risk and severity of MS. These findings inform the development of dietary-based strategies for MS prevention and treatment.

## 1 Introduction

Multiple sclerosis (MS) is a chronic inflammatory demyelinating disease of the central nervous system, affecting over 2.9 million people worldwide (1). MS is one of the most common causes of disability in young individuals, with onset typically occurring between the ages of 18–40 years. The overall female-to-male ratio is 3:1 (2). MS is characterized by multifocal demyelination and neuronal injury, resulting in a wide range of clinical symptoms including motor, sensory, visual, and cognitive impairments, as well as fatigue (2). While the exact etiology of MS remains uncertain, observational studies and emerging pathophysiology suggest that interactions between environmental and lifestyle factors with susceptible genes are the main mechanisms underlying the development of MS (2). Environmental factors not only influence the occurrence of MS but also impact its course and progression. Common environmental factors include smoking, viral infections (e.g., Epstein–Barr virus infection), sunlight exposure (living farther from the equator), vitamin D deficiency (including low intake and low serum concentrations), obesity, and dietary habits (2, 3). Extensive research indicates that nutritional and dietary factors influence the pathological mechanisms, development, and activity of MS through various pathways, including metabolism, inflammation, and the gut-brain axis (4, 5).

Current research on nutrition and diet in relation to MS encompasses various aspects, including the study of the effects of specific foods and nutrients on the development and progression of the disease. Additionally, therapeutic diets, such as dietary supplementation, are being explored as potential interventions to alleviate the progression of MS (4, 6, 7). Recent observational studies have found that the anti-inflammatory diet such as the Mediterranean diet is associated with a lower risk of developing MS and lower patient-reported disability (8, 9). The Mediterranean diet is characterized by high consumption of whole grains, fruits, vegetables, olive oil, and fish, with limited processed foods and red meat intake. Studies have found an association between increased pork consumption and increased MS risk, while cereal/bread and fish intake are associated with a reduced MS risk (10, 11). However, a study found that the consumption of unprocessed red meat may have a protective effect against central nervous system demyelination in women (12). Due to the influence of various confounding factors in observational studies, several clinical trials have been conducted to investigate the effects of dietary supplements (such as fish oil and various vitamins) on MS symptoms and progression. A double-blind clinical study found that supplementation with vitamin B12 and folic acid improved the quality of life in MS patients (13). A systematic review of seven clinical trials concluded that fish oil supplementation is beneficial for reducing relapse rates, inflammatory markers, and improving the quality of life in MS patients (14). However, current research suffers from small sample sizes, inconsistent findings, and lacks systematic studies specifically focusing on dietary components related to the severity of MS. Given the potential beneficial effects of diet on MS, larger-scale studies are urgently needed to systematically determine the role of different dietary components in preventing MS and improving prognosis.

With the emergence of large-scale genome-wide association studies (GWAS) on various dietary compositions, Mendelian randomization (MR) can help further explore the causal relationships between diet and disease (15–17). MR utilizes genetic variations randomly allocated during meiosis as instrumental variables to investigate the relationship between exposure and outcome, thereby reducing the risk of confounding factors or reverse causality compared to observational studies. MR may help further understanding the connection between diet and MS from a genetic perspective. Previous research has identified diet-related genes, such as the melanocortin 4 receptor involved in appetite regulation, as potential targets for reducing inflammation and slowing the progression of MS (18, 19). Populations with adaptive gene polymorphisms related to diet, originally selected based on their ancestral dietary contexts, are at an increased risk of obesity-related chronic diseases (20). Interestingly, previous MR studies have already revealed a causal link between obesity and MS (21). In addition, previous MR studies have explored the associations between circulating nutrient levels and MS risk (22, 23). However, there is currently no comprehensive MR study summarizing the relationships between different dietary components and MS risk and severity.

In this MR study, we collected large-scale GWAS data from the UK Biobank, GWAS and Sequencing Consortium of Alcohol and Nicotine Use (GSCAN), and International Multiple Sclerosis Genetics Consortium (IMSGC), and utilized a two-sample MR analysis to reveal the causal associations between 30 genetically predicted dietary compositions and the risk and severity of MS. This study aims to provide comprehensive, high-quality evidence for dietary interventions in individuals at high risk for MS and in MS patients.

## 2 Methods

### 2.1 Mendelian randomization design

**Figure 1:**
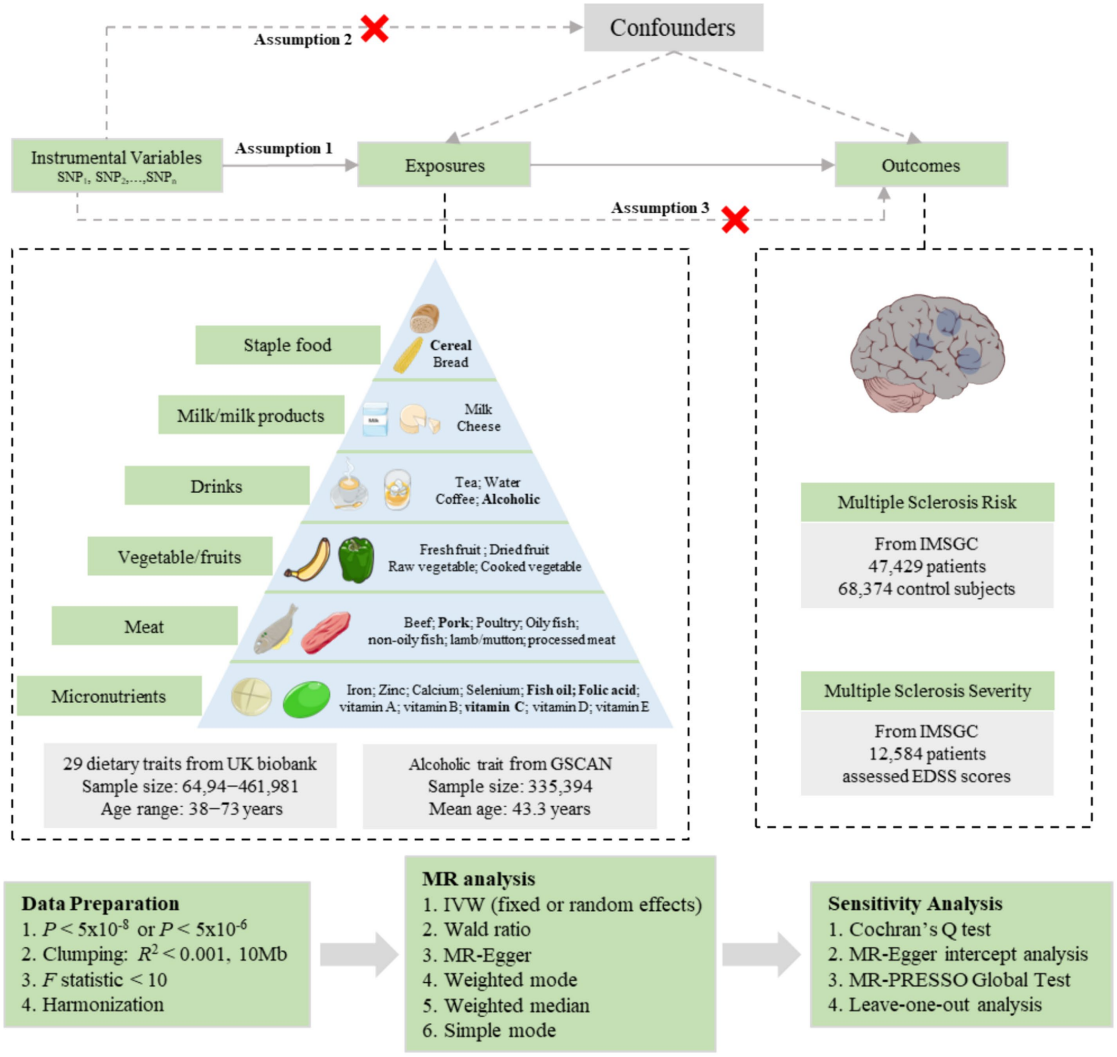
Schematic representation of the Mendelian randomization study on the causal relationship between dietary composition and multiple sclerosis risk as well as severity. SNP, single-nucleotide polymorphism; MR, Mendelian randomization; IVW, inverse variance weighted; GSCAN, GWAS and Sequencing Consortium of Alcohol and Nicotine Use; IMSGC, International Multiple Sclerosis Genetics Consortium. EDSS, Expanded Disability Status Scale. Some parts of the figure were illustrated using images from Servier Medical Art, provided by Servier and licensed under a Creative Commons Attribution 3.0 Unported License (https://creativecommons.org/licenses/by/3.0/).

In this study, we implemented a two-sample MR design to investigate the causal relationship between dietary composition and the risk and severity of MS, as illustrated in Figure 1. The datasets utilized in our study were obtained from publicly available databases and were subjected to approval by an ethics committee before their use. Therefore, no additional ethical approval was necessary for the conduct of this study. All the GWAS data included in these studies were sourced from European populations. We selected single nucleotide polymorphisms (SNPs) that are associated with exposures as instrumental variables (IVs). The MR design relies on three fundamental assumptions: (1) the genetic IVs should exhibit a strong association with the exposure variable; (2) the genetic IVs should be independent of potential confounding factors; and (3) the genetic IVs should solely influence the outcome through the exposure variable.

### 2.2 GWAS data sources

We collected the GWAS data for dietary composition from OpenGWAS^1^. A total of 30 different dietary compositions were selected as exposures and were divided into six categories, including staple food intake (cereal intake and bread intake), milk/milk products (milk intake and cheese intake), vegetable/fruits intake (raw vegetable intake, cooked vegetable intake, fresh fruit intake, and dried fruit intake), drinks intake (alcoholic intake, coffee intake, tea intake, and water intake), meat intake (beef intake, lamb/mutton intake, pork intake, oily fish intake, non-oily fish intake, poultry intake, and processed meat intake), and micronutrients/others supplement (vitamin A, vitamin B, vitamin C, vitamin D, vitamin E, folic acid, fish oil, calcium, iron, zinc, and selenium). Except for the alcoholic intake (alcoholic drinks per week) conducted by the GSCAN (*N* = 335,394) (24), the GWAS data for dietary composition were mainly conducted by the UK biobank (*N* = 64,949 − 461,981). Specific information regarding each exposure trait can be found in Figure 1 and Supplementary Table S1.

Summary statistics data for MS risk and severity was conducted by the IMSGC. Genetic data from 47,429 MS patients and 68,374 control subjects (including discovery and replication cohorts) were analyzed to conduct the GWAS data for MS risk, resulting in the identification of 200 autosomal susceptibility variants outside the major histocompatibility complex (MHC), one chromosome X variant, and 32 variants within the extended MHC (25). The discovery cohorts comprised data from 15 GWAS studies conducted in America, Europe, and Australia, with an approximate male proportion of 40.8%. The replication cohorts consisted of two large-scale replication cohorts, including 9 datasets (male: 29.6%) and 11 datasets (male: 42.3%) respectively. Principal covariates, such as age and sex, were adjusted for in the association tests. Detailed descriptions of contributing cohorts, including diagnosis criteria and demographic characteristics, were provided in the original publications. Additionally, the GWAS data for MS severity were obtained from a subset of the sample, specifically 12,584 MS cases recruited through 21 centers from North America, Europe, and Australia (26). These cases (male: 28.3%) had effectively declared their clinical outcome, with an average age at the last follow-up of 51.7 years and an average disease duration of 18.2 years. The neurological disability of these cases was assessed using the Expanded Disability Status Scale (EDSS) score. Most EDSS scores were assessed by neurologists, but in a small percentage (4.6%), questionnaires were used to approximate the scores. EDSS measures were adjusted for aging by using age-specific rankings to calculate the age-related MS severity score. More detailed information about the GWAS data included in this study can be found in Supplementary Table S1.

### 2.3 Selection of instrumental variables

We implemented a series of quality control measures to select suitable IVs for the MR analysis of the association between dietary composition and the risk and severity of MS. A significance threshold (*p* < 5 × 10^−8^) was used to identify SNPs with the strongest evidence of association. However, five dietary traits did not produce any instrumental variables in the final MR analysis, so their significance threshold was set to *p* < 5 × 10^−6^. Next, we performed a linkage disequilibrium (LD)-based clumping procedure with *R*^2^ < 0.001 and a window size of 10 Mb using the 1,000 Genomes EUR reference panel to ensure the independence of each IV in the MR analysis. The genetic IVs that showed associations with the risk or severity of MS at a significance level below 5 × 10^−8^ were excluded. We also assessed the IVs strength using the *F*-statistic (*F* = *β*^2^/se^2^) (27). We excluded SNPs with an *F*-statistic below 10, as they were considered weak IVs and could introduce bias into the analysis. To ensure consistency of effect alleles between the exposure and outcome, we performed SNP harmonization. This process involved eliminating ambiguous SNPs with intermediate allele frequencies and those with inconsistent alleles. Figure 1 depicts the IV selection process.

### 2.4 Statistical analyses

In this study, Wald’s ratio method (one IV available) and inverse variance weighted (IVW; more than one IVs available) method were used as the primary approach to estimate the causal effects of dietary composition on the risk and severity of MS. When the number of genetic IVs available was fewer than three, the fixed-effect IVW method was employed. In contrast, if there were more than three genetic IVs, the multiplicative random-effect IVW method was utilized (28). The IVW method combines meta-analysis principles with Wald estimation for individual SNPs, but it can only be utilized in scenarios without horizontal pleiotropy. To ensure the robustness of the MR results, MR-Egger, weighted median, weighted mode, and simple mode methods were also used to estimate the causal effects (29). The MR-Egger regression method assumes that more than 50% of the IVs are influenced by horizontal pleiotropy. If the intercept term is zero, the findings of MR-Egger regression are consistent with those of IVW, indicating the lack of horizontal pleiotropy. The weighted median method enables unbiased estimation of causal effects, even in scenarios where up to 50% of the IVs are invalid. The simple mode refers to an unweighted mode of the empirical density function used in causal estimation.

Several sensitivity analyses were performed to ensure the robustness of the MR results. We utilized Cochran’s *Q* test to identify heterogeneity using the IVW approach (30). The MR-Egger regression was employed to examine whether the results were influenced by directional horizontal pleiotropy (31). Moreover, we employed the Mendelian randomization pleiotropy residual sum and outlier (MR-PRESSO) method to identify and correct potential pleiotropy bias by detecting any outliers (32). Additionally, to assess the influence of individual SNPs, we conducted leave-one-out sensitivity analyses to validate the stability of the estimated causal effects when more than two IVs are available (15). We also conducted reverse MR analysis by treating the risk and severity of MS as the exposure with the IVs significance threshold of *p* < 5 × 10^−8^. This method involved excluding each SNP from the IVs to identify potential outliers. The statistical analysis was carried out using R software (version 4.1.3). The “TwoSampleMR” package (version 0.5.10) and the “MR-PRESSO” package (version 1.0) were employed for the MR analyses and MR-PRESSO analysis, respectively.

## 3 Results

Finally, we obtained a total of 692 IVs for 30 dietary compositions, with the number of IVs ranging from 9 to 124 for each composition. The range of the *F*-statistic values for each IV spans from 21 to 927, indicating the absence of weak IVs. Detailed information regarding the IVs for dietary composition can be found in Supplementary Table S2.

### 3.1 Causal effects of dietary composition on MS risk

**Figure 2:**
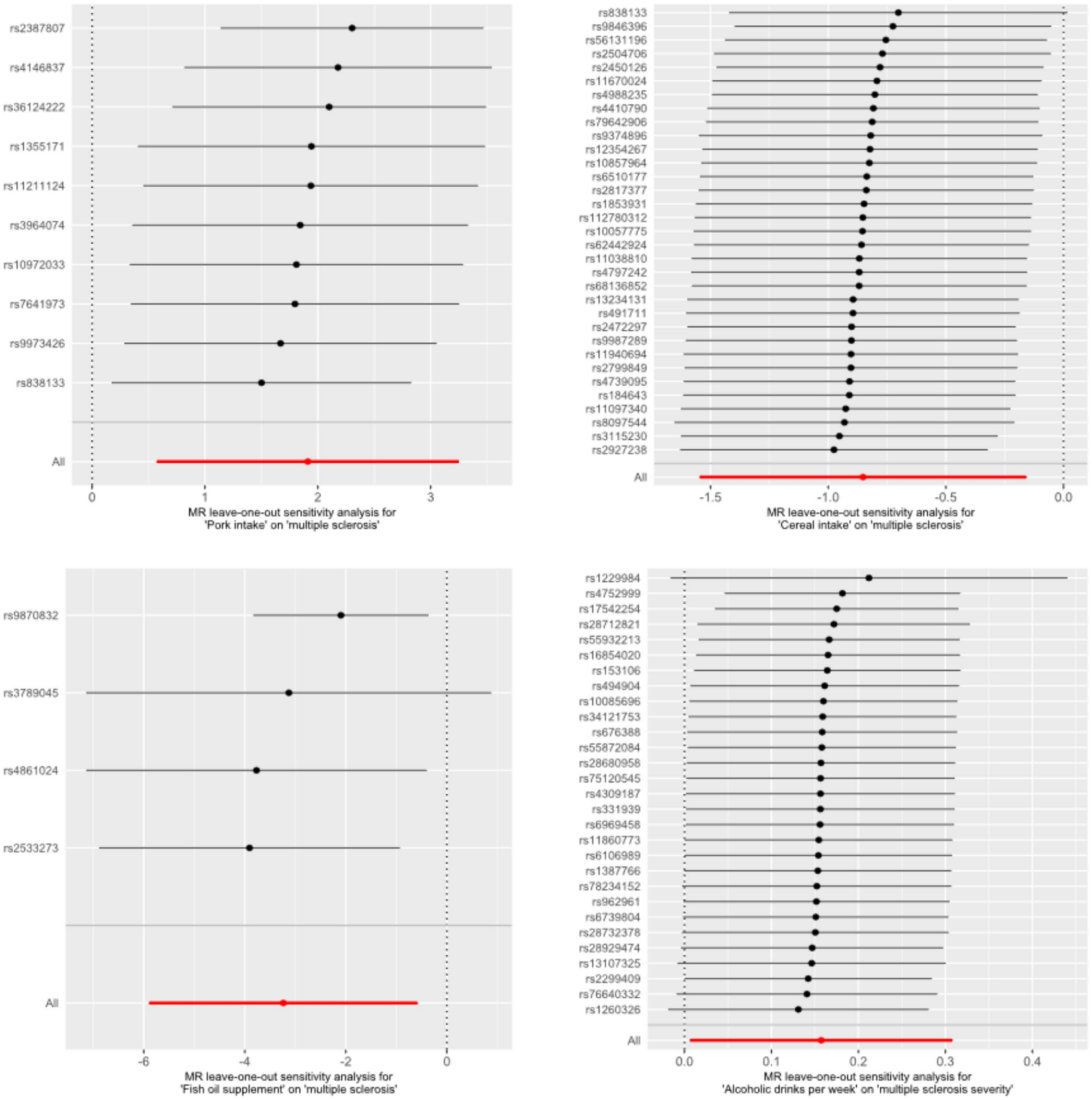
The results of a leave-one-out analysis.

The MR results of causal links between dietary composition and MS risk were found in Table 1 and Supplementary Table S3. In the MR analysis, five dietary intakes were found to be causally associated with the risk of MS. Specifically, genetically determined higher pork intake was observed to be associated with an increased risk of MS using the IVW method (odds ratio [OR] = 6.76; 95% CI = 1.77–25.81; *p* = 0.005). Similar results were obtained when using the weighted median method (*p* = 0.009). Conversely, individuals genetically predisposed to consuming higher quantities of cereals had a decreased risk of MS using the IVW method (OR = 0.43; 95% CI = 0.21–0.85; *p* = 0.016). Similar results were obtained when using the weighted median method (*p* = 0.037). Furthermore, based on the IVW method, genetically determined fish oil supplements (OR = 0.04; 95% CI = < 0.01–0.56; *p* = 0.017) were found to be causally linked to a reduced risk of MS. Based on the Wald ratio method, genetically determined intake of vitamin C supplement (OR < 0.01; 95% CI = < 0.01–< 0.01; *p*  = 4.34 × 10^−5^) and folic acid supplement (OR < 0.01; 95% CI = < 0.01–< 0.01; *p*  = 4.91 × 10-71) were observed to be causally linked to a reduced risk of MS. Only the association between cereal intake and MS risk presented potential heterogeneity (Cochran’s *Q* test *p* = 3.58 × 10^−4^; see Supplementary Table S4) and pleiotropy (MR-PRESSO global test *p* = 0.001; see Supplementary Table S5). However, after correcting the outliers, the causal effect of cereal intake on MS risk remained significant (outlier-corrected *p =* 0.008; see Supplementary Table S6). Reverse MR analysis revealed no reverse causal relationship between the aforementioned dietary compositions and MS risk (Supplementary Table S11). In the Leave-one-out analysis, no SNPs were identified as driving the association between the aforementioned dietary compositions and MS risk (Figure 2).

**Table 1:** MR results of causal links between dietary composition and multiple sclerosis risk.

**Class**	**Exposure**	**SNP**	***p*-value**	**OR (95% CI)**	***p*-value (Cochran’s *Q*)**	***p*-value** **(MR-Egger intercept)**	***p*-value** **(Global Test)**
**Staple food**	Cereal intake	33	0.016	0.43 (0.21, 0.85)	3.58E-04	0.167	0.001
	Bread intake	25	0.830	0.89 (0.32, 2.52)	1.56E-10	0.212	<0.001
**Milk/milk products**	Milk intake	11	0.716	1.26 (0.36, 4.45)	0.014	0.357	0.014
	Cheese intake	49	0.804	1.05 (0.70, 1.58)	0.021	0.188	0.004
**Drinks**	Alcoholic intake	29	0.603	0.88 (0.55, 1.41)	0.029	0.913	0.057
	Coffee intake	33	0.765	0.93 (0.58, 1.49)	0.237	0.177	0.044
	Tea intake	30	0.295	0.78 (0.49, 1.24)	0.016	0.466	0.04
	Water intake	34	0.461	0.74 (0.33, 1.65)	3.93E-09	0.952	<0.001
**Vegetable/fruits**	Raw vegetable intake	10	0.522	0.55 (0.09, 3.47)	0.012	0.112	<0.001
	Cooked vegetable intake	15	0.056	6.31 (0.95, 41.77)	7.80E-09	0.495	<0.001
	Fresh fruit intake	47	0.582	1.88 (0.20, 17.76)	1.91E-120	0.533	<0.001
	Dried fruit intake	35	0.084	0.64 (0.38, 1.06)	0.522	0.513	<0.001
**Meats**	Beef intake	11	0.487	0.62 (0.16, 2.41)	0.011	0.687	0.01
	Lamb/mutton intake	26	0.819	1.11 (0.44, 2.82)	0.012	0.182	0.002
	Pork intake	10	0.005	6.76 (1.77, 25.81)	0.181	0.703	0.102
	Oily fish intake	48	0.144	0.65 (0.36, 1.16)	1.58E-07	0.430	<0.001
	Non-oily fish intake	11	0.064	3.3 (0.93, 11.64)	0.041	0.031	0.039
	Poultry intake	7	0.909	0.91 (0.18, 4.71)	0.075	0.501	0.099
	Processed meat intake	17	0.410	1.41 (0.62, 3.18)	0.025	0.557	0.024
**Micronutrients/others**	Vitamin A	1	0.192	<0.01 (<0.01, >100)	–	–	–
	Vitamin B	8	0.964	1.13 (0.01, >100)	0.616	0.670	0.704
	Vitamin C	1	4.34E-05	<0.01 (<0.01, <0.01)	–	–	–
	Vitamin D	8	0.261	<0.01 (<0.01, 92.73)	0.003	0.217	0.012
	Vitamin E	7	0.056	>100 (0.85, >100)	0.492	0.907	0.567
	Folic acid or Folate	1	4.91E-71	<0.01 (<0.01, <0.01)	–	–	–
	Fish oil	4	0.017	0.04 (<0.01, 0.56)	0.399	0.800	0.482
	Calcium	3	0.433	9.81 (0.03, >100)	0.335	0.410	–
	Iron	1	0.417	<0.01 (<0.01, >100)	–	–	–
	Zinc	3	0.937	1.37 (0.04, >100)	0.811	0.654	-
	Selenium	7	0.077	>100 (0.36, >100)	0.251	0.944	0.315

MR, Mendelian randomization; OR, odd ratio; CI, confidence interval; SNP, single-nucleotide polymorphism.

### 3.2 Causal effects of dietary composition on MS severity

The MR results of causal links between dietary composition and MS severity were found in Table 2 and Supplementary Table S7. Only two dietary intakes were found to be causally associated with the risk of MS severity. Specifically, genetically predicted higher alcoholic intake was causally associated with an increase in MS severity using the IVW method (OR = 1.17; 95% CI = 1.01–1.36; *p* = 0.041). Conversely, genetically determined intake of folic acid supplements was causally linked to a decrease in MS severity using the IVW method (OR < 0.01; 95% CI = < 0.01–0.09; *p* = 0.015). In the sensitivity analyses, no potential heterogeneity and pleiotropy was found in these significant associations (Table 2 and Supplementary Tables S8–S10). MR analysis revealed no reverse causal relationship between the aforementioned dietary compositions and MS severity (Supplementary Table S11). In the Leave-one-out analysis, no SNPs were identified as driving the association between the aforementioned dietary compositions and MS severity (Figure 2).

**Table 2:** MR results of causal links between Dietary Composition and multiple sclerosis severity.

**Class**	**Exposure**	**SNP**	***p*-value**	**OR (95% CI)**	***p*-value (Cochran’s *Q*)**	***p*-value** **(MR-Egger intercept)**	***p*-value** **(Global Test)**
**Staple food**	Cereal intake	34	0.559	0.93 (0.71, 1.20)	0.943	0.768	0.973
	Bread intake	27	0.302	1.24 (0.82, 1.87)	0.063	0.483	0.163
**Milk/milk products**	Milk intake	20	0.982	1.00 (0.67, 1.51)	0.646	0.388	0.679
	Cheese intake	54	0.248	1.14 (0.91, 1.41)	0.574	0.352	0.696
**Drinks**	Alcoholic intake	29	0.041	1.17 (1.01, 1.36)	0.969	0.906	0.968
	Coffee intake	35	0.374	1.13 (0.86, 1.48)	0.524	0.137	0.616
	Tea intake	32	0.957	0.99 (0.77, 1.28)	0.264	0.857	0.255
	Water intake	36	0.343	0.85 (0.61, 1.19)	0.140	0.152	0.269
**Vegetable/fruits**	Raw vegetable intake	11	0.827	0.92 (0.42, 2.01)	0.461	0.422	0.852
	Cooked vegetable intake	15	0.854	1.06 (0.57, 1.97)	0.392	0.282	0.463
	Fresh fruit intake	49	0.311	1.26 (0.81, 1.95)	0.126	0.372	0.035
	Dried fruit intake	35	0.903	0.97 (0.64, 1.48)	0.105	0.669	0.297
**Meats**	Beef intake	12	0.580	1.15 (0.70, 1.90)	0.760	0.811	0.710
	Lamb/mutton intake	26	0.262	0.74 (0.44, 1.25)	0.274	0.031	0.328
	Pork intake	11	0.703	0.85 (0.36, 1.98)	0.239	0.229	0.321
	Oily fish intake	52	0.096	0.83 (0.66, 1.03)	0.840	0.400	0.779
	Non-oily fish intake	11	0.058	0.59 (0.34, 1.02)	0.679	0.530	0.669
	Poultry intake	7	0.167	1.68 (0.80, 3.51)	0.597	0.591	0.428
	Processed meat intake	21	0.565	0.89 (0.61, 1.31)	0.455	0.811	0.293
**Micronutrients/others**	Vitamin A	1	0.321	>100 (<0.01, >100)	–	–	–
	Vitamin B	13	0.708	0.61 (0.04, 8.34)	0.765	0.789	0.867
	Vitamin C	1	0.077	0 (<0.01, 1.99)	–	–	–
	Vitamin D	11	0.884	0.75 (0.02, 33.46)	0.373	0.974	0.271
	Vitamin E	10	0.214	0.03 (<0.01, 7.39)	0.102	0.176	0.014
	Folic acid or Folate	2	0.015	<0.01 (<0.01, 0.09)	0.313	–	–
	Fish oil	4	0.423	2.1 (0.34, 12.83)	0.422	0.470	0.486
	Calcium	4	0.323	13.78 (0.08, >100)	0.097	0.334	0.181
	Iron	1	0.545	33.44 (<0.01, >100)	–	–	–
	Zinc	3	0.083	>100 (0.52, >100)	0.769	0.855	–
	Selenium	7	0.841	1.97 (<0.01, >100)	0.221	0.066	0.306

MR, Mendelian randomization; OR, odd ratio; CI, confidence interval; SNP, single-nucleotide polymorphism.

## 4 Discussion

In this study, we applied a two-sample MR method to detect the causal effects of 30 dietary compositions on the risk and severity of MS. Finally, five dietary compositions associated with MS risk and two dietary compositions linked to MS severity were identified. Specifically, we found that genetically predicted higher pork intake may increase MS risk, while genetically driven higher cereal intake, vitamin C supplements, folic acid supplements, and fish oil supplements may reduce MS risk. Furthermore, genetically predicted higher alcohol intake may increase MS severity, while folic acid supplements may decrease MS severity.

Nutrition and dietary factors, as well as dietary interventions, have long been recognized as important etiological mechanisms of MS and complementary treatments in controlling disease progression, respectively (4, 6, 7). It is currently believed that dietary factors involved in MS pathogenesis may be related to inflammation-associated pathways such as oxidative stress (5). Recent research has emphasized the role of proinflammatory diets in the development of MS (5). High intake of red meat and alcohol falls under the category of a proinflammatory diet. Our study found that genetically predicted higher pork intake is causally associated with an increased risk of MS, which is supported by previous observational studies. As early as 1986, a study found a significant correlation between pork consumption and MS incidence (*R* = 0.87) (10). Another Canadian cohort study found an association between pork intake and increased MS risk (OR = 1.24) (11). However, a study conducted in Australia revealed that higher consumption of unprocessed red meat was associated with a 26% reduction in the risk of central nervous system demyelination in females (12). Red meat contains arachidonic acid, which activates Th17 cells involved in inflammatory pathways. Additionally, red meat promotes the formation of nitrosamines, thereby facilitating chronic inflammation (33). Furthermore, we also found that increased alcohol intake is associated with increased severity of MS. Alcohol consumption is prevalent among MS patients (34), but there have been inconsistent findings regarding the association between alcohol and MS risk. Recently, a large cohort study using the UK Biobank dataset and previous meta-analyses did not find an increased risk of MS with alcohol consumption, which is consistent with our study results (35, 36). However, a Danish cohort study found that alcohol consumption in adolescence was associated with a lower risk of developing MS in both sexes (37). There are also inconsistent findings regarding whether alcohol consumption affects disease progression or severity. An Italian cohort study found no association between alcohol intake and MS severity, unlike smoking (38). Conversely, a follow-up study in Mexico revealed that ex-consumers of alcohol had a lower risk of MS progression than current consumers (39). In conclusion, the relationship between alcohol and MS is still uncertain and requires further in-depth research, such as distinguishing the types of alcohol consumed and its relationship with different MS subtypes (40).

Our study emphasizes the protective role of an anti-inflammatory diet in MS. We found a high intake of cereals and increased supplementation of fish oil were associated with a lower risk of MS. These findings are supported by previous research. As early as 1998, a Canadian case–control study found a correlation between cereal/bread intake and a lower risk of MS (OR = 0.62) (11). However, it is important to note that some studies suggest that gluten may impact the progression of MS (41). Fish oil, rich in omega-3 polyunsaturated fatty acids, such as eicosapentaenoic acid and docosahexaenoic acid, may have anti-inflammatory, antioxidant, and neuroprotective effects (42). A systematic review revealed the beneficial effects of fish oil supplementation and omega-3 fatty acids in improving quality of life and reducing relapse rates and inflammation markers in MS patients (14). A study found that 1 year of fish oil consumption can increase mitochondrial membrane fluidity and decrease ATP hydrolase activity, leading to neuroprotection, antioxidant, and anti-inflammatory effects in relapsing–remitting MS (43). Additionally, supplementation of fish oil in MS patients during remission can effectively lower levels of cytokines (such as TNF-α, IL-1β, IL-6) and nitric oxide metabolites (42). However, another study shows negative results (44).

Numerous studies have explored the relationship between various vitamins and MS, with vitamin D being the most extensively studied. An MR study suggests a causal relationship between circulating vitamin D levels and the risk of MS. However, our study found that increased supplementation of vitamin D did not reduce the risk of MS or the severity of the disease. The latter finding is supported by previous research. A systematic review conducted by Cochrane in 2018 found no significant benefits of vitamin D supplementation in terms of any form or dosage for MS patients (45). Therefore, the current evidence regarding the supplementation of vitamin D for the prevention or improvement of MS prognosis remains insufficient and requires confirmation through larger sample studies.

It is worth noting that our study found that a higher folic acid supplement not only reduces the risk of MS but also lowers the severity of the disease. Additionally, we revealed that higher vitamin C supplements can decrease the risk of MS. A case–control study involving 143 cases of primary progressive MS found that nutrient supplementation with calcium, iron, folic acid, vitamin B12, and C was associated with more than an 84% lower risk of MS (46). A double-blind clinical study revealed that supplementation with vitamin B12 and folic acid can lower the mean homocysteine levels in MS patients and improve quality of life (13). However, studies investigating the association between circulating vitamin levels and MS have yielded inconsistent conclusions. A follow-up study involving 140 MS patients found that low circulating folate levels at 8 months of follow-up were associated with increased disease severity (47). Two MR studies found no association between circulating folate or vitamin C levels and the risk of MS. The discrepancy in results between nutrient supplementation and circulating nutrient levels could potentially be explained by the fact that nutrients may influence pathophysiological processes through other indirect pathways, such as affecting the gut microbiota (48).

A connection between vegetables/fruits and MS was not found in our research. It has been observed in several studies that vegetable/fruit intake may serve as a protective factor for MS (49). However, it should be noted that many studies have examined mixed dietary patterns, such as a vegetable/low-protein diet or plant-based diet (49, 50). A recent study has suggested that a vegetarian diet may be not associated with a decreased risk of MS (8). Regarding milk intake and MS, our study did not find evidence supporting a clear association. It appears to be a more complex issue. A study conducted in Australia, a country with moderate to low latitudes, found no association between milk intake and the first diagnosis of demyelination (51). Additionally, a retrospective case–control study involving 536 Iranian MS patients found that the frequency of milk intake was higher in the healthy control group compared to the MS group (52). On the contrary, in Denmark, a country with high latitudes, the risk of developing MS was found to be doubled in dairy workers compared to the general population over a 10-year period (53). It is worth noting that the GWAS data we included for MS came from multiple countries, including Europe and Australia, so future research may require subgroup analysis of GWAS studies for MS in different populations.

Our study has some limitations. Firstly, we only have access to phenotype GWAS data for MS risk and severity, thus limiting our ability to explore the relationship between diet and disease progression, different clinical subtypes, and specific symptoms of MS using MR (54). Future studies should incorporate GWAS data for different clinical subtypes of MS, as prognosis varies among these subtypes. In addition, MS is a multifactorial disease that requires considering the effects of genetic and environmental confounding factors such as age, gender, and distance to the equator. Future research exploring the impact of dietary factors on MS in different populations may effectively control these confounders. Secondly, it is worth noting that our study only assessed the isolated effects of single nutrients on MS risk and severity, which can present challenges when making dietary recommendations. Therefore, our findings should be interpreted with caution. Before making dietary recommendations, it is essential to consider individual variations and the overall dietary structure. Exploring overall dietary patterns, such as the Mediterranean Diet, Paleolithic Diet, McDougall Diet, etc., may have more practical research value (5). Previous studies have indicated that the Mediterranean Diet is associated with a lower risk of developing MS and lower patient-reported disability (8, 9). However, the existence of genetic variations related to different dietary patterns lacks relevant GWAS research, which could be an important future research direction. Thirdly, the GWAS data used in this study primarily consist of individuals of European ancestry, which may limit the generalizability of our findings to other ethnic populations.

## 5 Conclusion

In conclusion, our MR study showed that increasing intake of cereal, vitamin C, folate, and fish oil, while reducing consumption of pork and alcohol, may potentially lower the risk and severity of MS. These findings may provide information for the development of MS prevention strategies and disease-modifying treatments based on dietary adjustments, especially the anti-inflammatory diet. Further large-scale clinical trials are needed to confirm these findings.

## Data availability statement

Publicly available datasets were analyzed in this study. This data can be found here: https://gwas.mrcieu.ac.uk/. The names of the repository/repositories and accession number(s) can be found in the article/Supplementary material. The original contributions presented in this study are included in the article/Supplementary material, further inquiries can be directed to the corresponding authors.

## Author contributions

HN: Conceptualization, Formal analysis, Funding acquisition, Methodology, Writing – original draft, Writing – review & editing.

## Footnotes

^1^ https://gwas.mrcieu.ac.uk/
